# FoxM1 transactivates PTTG1 and promotes colorectal cancer cell migration and invasion

**DOI:** 10.1186/s12920-015-0126-9

**Published:** 2015-08-12

**Authors:** Yun Zheng, Jinjun Guo, Jin Zhou, Jinjian Lu, Qi Chen, Cui Zhang, Chen Qing, H. Philip Koeffler, Yunguang Tong

**Affiliations:** Department of Medicine, Cedars-Sinai Medical Center, UCLA School of Medicine, Room 3021, 8700 Beverly Blvd, Los Angeles, CA 90048 USA; Sun Yat-sen University Cancer Center; State Key Laboratory of Oncology in South China, Collaborative Innovation Center for Cancer Medicine, Guangzhou, China; Department of Gastroenterology and Hepatology, The Second Affiliated Hospital of Chongqing Medical University, Chongqing, China; Division of Epidemiology and Biostatistics, College of Public Health, University of Arizona, Tucson, AZ USA; State Key Laboratory of Quality Research in Chinese Medicine, Institute of Chinese Medical Sciences, University of Macau, Macao, China; Department of Pathology, Xinxiang Medical University, 601 East Jinsui Ave, Xinxiang, Henan China; School of Pharmaceutical Science, Kunming Medical University, 1168 Western Chunrong Road,Yuhua Street, Chenggong New City, Kunming China

## Abstract

**Background:**

Metastasis is the major cause of cancer-related death. Forkhead Box M1 (FoxM1) is a master regulator of tumor metastasis. This study aims to identify new FoxM1 targets in regulating tumor metastasis using bioinformatics tools as well as biological experiments.

**Methods:**

Illumina microarray was used to profile WT and PTTG1 knockout HCT116 cells. R2 Genomics Analysis was used to identify PTTG1 as a potential FoxM1 targeted gene. Luciferase reporter array, EMSA and Chromatin Immunoprecipitation (ChIP) were used to determine the binding of FoxM1 to PTTG1 promoter. Boyden chamber assay was used to evaluate the effects of FoxM1-PTTG1 on cell migration and invasion. Splenic-injection induced liver metastasis model was used to evaluate the effects of FoxM1-PTTG1 on liver metastasis of colorectal cancer.

**Results:**

Analyses of multiple microarray datasets derived from human colorectal cancer indicated that correlation levels of FoxM1 and pituitary tumor transforming gene (PTTG1) are highly concordant (R = 0.68 ~ 0.89, *p* = 2.1E-226 ~ 9.6E-86). FoxM1 over-expression increased and knock-down decreased PTTG1 expression. Luciferase reporter assay identified that the −600 to −300 bp region of PTTG1 promoter is important for FoxM1 to enhance PTTG1 promoter activity. EMSA and ChIP assays confirmed that FoxM1 directly binds to PTTG1 promoter at the −391 to −385 bp region in colorectal cancer cells. Boyden chamber assay indicated that both FoxM1 and PTTG1 regulate migration and invasion of HCT116 and SW620 colorectal cancer cells. Further *in vivo* assays indicated that PTTG1 knock out decreased the liver metastasis of FoxM1 over-expressing HCT116 cells. Microarray analyses identified 662 genes (FDR < 0.05) differentially expressed between WT and PTTG1^−/−^ HCT116 cells. Among them, dickkopf homolog 1 (DKK1), a known WNT pathway inhibitor, was suppressed by PTTG1 and FoxM1.

**Conclusions:**

PTTG1 is a FoxM1 targeted gene. FoxM1 binds to PTTG1 promoter to enhance PTTG1 transcription, and FoxM1-PTTG1 pathway promotes colorectal cancer migration and invasion.

**Electronic supplementary material:**

The online version of this article (doi:10.1186/s12920-015-0126-9) contains supplementary material, which is available to authorized users.

## Background

Colorectal cancer (CRC) is the third most commonly diagnosed cancer and the second leading cause of cancer-related death in the United States. Almost a third of CRC patients have metastatic disease at diagnosis [1]. Half of the patients diagnosed and resected with early-stage disease subsequently develop metastasis [2]. The process of cancer metastasis consists of a series of sequential and interrelated steps, including invasion, detachment from the primary sites, intravasation, survival in the circulation and translocation to microvessels of target organs, extravasation and colonization [3]. Several pathways, including Cadherin-catenin system, integrins, matrix metalloproteinases (MMPs), epidermal growth factor receptor (EGFR) signaling pathways (including the RAS-RAF-MAPK and PI3K/AKT), and the c-Met and hepatocyte growth factor/scatter factor (HGF/SF) signaling cascade (including the Wnt pathway, the Ras pathway and the PI3K/AKT pathway) have shown being involved in the CRC metastasis [4–9]. However, we still know very little about the exact molecular mechanisms that regulate CRC metastasis.

Pituitary tumor-transforming gene-1 (PTTG1) was isolated from rat pituitary tumor cells in 1997 [10]. As a vertebrate securin, PTTG1 functions in cell replication, cell cycle control, DNA damage/repair, organ development, metabolism, cell transformation and cell senescence [11, 12]. And more importantly, PTTG1 is highly expressed in tumors derived from a variety of organs, including colon, pituitary, thyroid, breast, ovary, uterine, lung and esophagus [13–19]. PTTG1 has been identified as a key signature gene associated with tumor metastasis. PTTG1 activates c-Myc and cyclin D3 (CCND3) to facilitate cell proliferation, increases basic fibroblast growth factor (bFGF), vascular endothelial growth factor (VEGF) and matrix metalloproteinase 2 (MMP2) expression to induce angiogenesis, which play an important role in tumor development and cancer metastasis, and also induces interleukin-8 to function in metastasis [11, 20–22]. PTTG1 interacts with transcription factors including p53, Sp1, and upstream stimulatory factor 1 (USF1), which may induce additional genes involved in tumorigenesis and cancer development [20, 23, 24]. In terms of colorectal cancer, PTTG1 overexpression is associated with tumourigenesis, progression and cancer metastasis [25].

The human forkhead box protein M1 (FoxM1) gene, consisting 10 exons, is mapped to chromosome 12p13-3, and plays important roles in cellular proliferation and differentiation during embryogenesis and development of cancer [26–28]. FoxM1 protein has been identified as a key regulator of cell cycle by targeting genes involved in G1/S progression and G2/M transition such as cyclin B1, Cdc25B, Aurora B and Polo like kinases, etc. [27]. Expression of FoxM1 is dramatically elevated in tumor cells derived from liver, lung, colon, breast and prostate [29]. FoxM1 contributed to the development and growth of mouse CRC [30] as well as a subset of human CRC [31]. FoxM1 is regarded as a master regulator of tumor metastasis [32]. Abnormal activation of FoxM1 leads to overexpression of multiple angiogenic genes, such as MMP-2, Cav-1, ZEB1 and ZEB2, which result in overexpression of multiple pro-invasion and -metastasis molecules [28, 33–35].

As described above, abnormal transcription and expression of PTTG1 and FoxM1 are involved in cancer progression and metastasis. However, the precise roles of PTTG1 and FoxM1 in CRC progression and metastasis remain unclear. In the present study, we demonstrated that FoxM1 and PTTG1 were concordantly up-regulated in colorectal cancers. FoxM1 activates PTTG1 and promotes migration and invasion of colorectal cancer cells.

## Methods

### Microarray data analyses

We used R2 Genomics Analysis and Visualization Platform (http://r2.amc.nl) to determine the correlation between FoxM1 and PTTG1 expression in seven microarray datasets derived from tumors. Six datasets were from Gene Expression Omnibus (GEO, http://www.ncbi.nlm.nih.gov/gds/), including three colon cancer datasets (GSE2109, *n* = 315; GSE41258, *n* = 388; GSE39582, *n* = 564), one breast cancer dataset (GSE3494, *n* = 251), one ovarian cancer dataset (GSE9891, *n* = 285) and one neuroblastoma dataset (GSE45547, *n* = 649). One glioma dataset (*n* = 403) was from TCGA. These datasets has been processed and stored in the server of R2 platform.

To identify potential PTTG1-targeted genes, total RNA from PTTG1^+/+^ and PTTG1^−/−^ HCT116 cells was isolated using the RNeasy Kit (Qiagen, Germantown, MD, USA) and analyzed using Illumina microarrays (HumanHT-12_V4). Raw data were processed using the manufacturer’s standard protocol and further analyzed using Partek 6.5. Microarray data have been deposited into the GEO database (GSE58863).

### Cell culture and transfections

HCT116 and SW620 cells were obtained from the American Tissue Culture Collection (ATCC, Manassas, VA, USA) and cultured at recommended conditions. HCT116 PTTG^+/+^ and PTTG^−/−^ cells were kindly provided by Dr Bert Vogelstein, Johns Hopkins University (Baltimore, MD, USA). Transfections were performed in 70–80 % confluent cells using Lipofectamine 2000 (Invitrogen, Grand Island, NY, USA) according to the manufacturer’s protocol.

### Plasmids and siRNAs

Four different human PTTG1 promoter fragments (from-1300 to +50, from −900 to +50, from −600 to +50 and from −300 to +50 bp ) were amplified from human genomic DNA (Roche Company, Basel, Switzerland) using TaKaRa LA Taq, and inserted into the pGL3-Basic luciferase reporter vector (Promega, Madison, WI, USA). Wild-type FoxM1 and PTTG1 expression plasmids were amplified from human FoxM1 and PTTG1 cDNA using a High-Fidelity polymerase (Clontech, Mountain View, CA, USA) and cloned into pcDNA3.1/myc-his vector (Invitrogen). All plasmids above were sequenced by Sequetech (Mountain View, CA, USA). Predesigned human PTTG1 siRNA (ID s17655), FoxM1 siRNA (ID s5248) and negative control No. 1 siRNA (Cat# AM4611) were obtained from Ambion (Grand Island, NY, USA).

### RNA extraction and real-time PCR

Total RNA was isolated using TRIZOL Reagent (Invitrogen). Three micrograms of total RNA were used to synthesize cDNA with SuperScript II Reverse Transcriptase (Invitrogen). Real-time PCR was amplified in 20 μl reaction mixtures (100 ng template, 0.5 μM of each primer, 10 μl 2× SYBR GREEN Master Mix (Applied Biosystems, Foster City, CA, USA) using the following parameters: 95 °C for 1 min, followed by 40 cycles of 95 °C for 20 s, 60 °C for 40 s. β-actin was used as internal control. Sequence of real-time PCR primers were provided in Additional file 1.

### Western blot analysis

Total cell lysate was prepared in RIPA buffer (Sigma, St. Louis, MO, USA) containing Protease Inhibitor Cocktail (Sigma). Protein concentrations were measured by Coomassie Plus Assay Kit (Pierce, Rockford, IL USA) using BSA as standard. Equal amounts (100 μg) of proteins were separated by NuPAGE Novex Bis-Tris Gels (Invitrogen) and transferred onto polyvinylidene difluoride (PVDF) membrane (Millipore, Billerica, MA, USA). The membrane was incubated in TBS buffer containing 5 % nonfat dry milk (Bio-Rad, Hercules, CA, USA) for 1 hour at room temperature, followed by incubation with the primary antibodies at 4 °C overnight: FoxM1 (1:200), PTTG1 (1:500) and DKK1 (1:200) were from Abcam (Cambridge, MA, USA). β-actin (1:5000) was from Sigma. After washes with TTBS (0.5 % Tween-20 in TBS), membranes were subsequently incubated with horseradish peroxidase linked secondary antibody (GE Healthcare, Piscataway, NJ, USA) for 1 hour at room temperature and developed using ECL Western Blotting Detection Reagents (GE Healthcare). Detected bands were quantified using Image J v1.43 as instructed in the software manual.

### Luciferase reporter assay

Cells were plated into 24-well plates and incubated at 37 °C overnight. Each well was co-transfected with: 1) 200 ng PTTG1 luciferase promoter, pGL3-Basic as control; 2) 800 ng FoxM1 expression plasmid, empty pcDNA3.1 vector as control; or 75 ng FoxM1 siRNA, negative control No. 1 siRNA as control; 3) pRL-TK (Promega) encoding Renilla luciferase was used as an internal control (10 ng/well) to assess transfection efficiency. After 24 hours, whole-cell lysates prepared with passive lysis buffer were collected for reporter detection by the Dual Luciferase Reporter System (Promega) according to the manufacturer’s protocol. Reactions were measured using an Orion Microplate Luminometer (Berthold Detection System, Pforzheim, Germany). Transfections were performed in triplicate and repeated four times to assure reproducibility.

### Electrophoresis mobility shift assay (EMSA)

Fluorescence-based Electrophoretic Mobility Shift Assay (EMSA) Kit (Invitrogen) was used to assess binding of FoxM1 on PTTG1 promoter. PTTG1 promoter 60 bp fragment (ATATTTC***TATTTAT***TTTCCATCCTTTTTACAGGGTCATC ***TAAATAA***AAATATCTTAAAGC, −398 to −339 bp upstream from the human PTTG1 TSS) was found containing two putative FoxM1 binding sites (−391 to −385 and −359 to −353 bp). The wild type fragment (defined as Biotin-Probe and a mutant without both FoxM1 binding sites (ATATTTC***gatcgtg***TTTCCATCCTTTTTACAGGGTCATC***atccttg***AAATATCTTA AAGC, defined as Biotin-dmProbe), and −391 to −385 mutant 60 bp probe (ATATTTC***gatcgtg***TTTCC ATCCTTTTTACAGGGTCATC***TAAATAA***AAATATCTTAAAGC, defined as 1^st^ site mProbe) were synthesized with 3’-ends labeled with biotin. The oligonucleotides without biotin were used as negative control and competitors, including wild type 60 bp probe (defined as Cold Probe), −398 to −377 wild type 22 bp fragment (ATATTTC***TATTTAT***TTTCCATC, defined as 1^st^ site oligo) and −367 to −363 wild type 25 bp fragment (GGGTCATC ***TAAATAA***AAATATCTTA, defined as 2^nd^ site oligo). FoxM1 was overexpressed in HCT116 cells, and nuclear protein was extracted using a nuclear extract kit (Active Motif). Protein concentration was measured by Coomassie Plus Assay Kit (Pierce). Oligonucleotides (1 μg) was incubated with 1 mg nuclear proteins for 20 min at room temperature in binding buffer containing 12 % glycerol, 12 mM HEPES (pH 7.9), 4 mM Tris (pH 7.9), 150 mM KCl, 1 mM EDTA, 1 mM dithiothreitol, and 10 μg polydeoxyinosinic deoxycytidylic acid competitor. The mixtures were analyzed according to the provided manual of the EMSA kits.

### Chromatin immunoprecipitation (ChIP)

Using a ChIP kit (Active Motif, Carlsbad, CA, USA), about 10^7^ cells were cross-linked and lysed. Chromatin was sheared to 300 to 700 bp fragments. Sheared chromatin DNA mixture (normalized inputs) was incubated with 4 μg FoxM1 antibody (#Santa Cruz Biotechnology, Santa Cruz, CA, USA) overnight at 4 °C. Negative control IgG and positive control H3 antibody were added at 10 μl (4 μg) per ChIP reaction. Real-time PCR were amplified using PTTG1 promoter primer (targeting −391 to −385 bp region), PTTG1 exon2 primer (as negative control) and VEGF promoter primer (as positive control).

### In vitro migration and invasion assay

Cell migration and invasion assays were performed in 6.5 mm Transwells (Corning #3422, Corning, NY, USA). Cells (2 × 10^5^) suspended in 100 μ l serum-free medium were added to the upper chamber and the lower chamber was filled with complete medium with 10 % serum. Cells were allowed to migrate at 37 °C for 48 h. After removing non-migrated cells, membranes were fixed in methanol and stained with 0.05 % crystal violet. Migrated cells were photographed and quantified in five random fields per membrane. Each sample was assayed in triplicate. For invasion assay, the Transwells were first coated with Matrigel and cells were allowed to invade for 72 h. For wound assay, cells were grown to confluence on 35 mm dishes. A wound was made in each cell monolayer. Images were captured on time 0 and 24 h. Cell migration distance was determined by subtracting values obtained at 0 h from 24 h. Migration distances were expressed as percentages over control values.

### Intrasplenic injection-induced liver metastases

Mouse injections were performed in accordance with the NIH Guide for the Care and Use of Laboratory Animals and approved by the Institutional Animal Care and Use Committee of Cedars-Sinai Medical Center. Briefly, Six-week-old female nude (nu/nu) mice (Jackson Laboratories) were anesthetized (by IP injecting Ketamine [75 mg/kg] and Dexmedetomidine [0.5 mg/kg] ). Carprofen at 5.0 mg/kg were injected subcutaneously after anesthesia induction and prior to performing surgery. The abdomen was disinfected with Iodine and alcohol swab and a small midline incision was made. The spleen is exteriorized and 10 cells in 50 μ l PBS were injected. After injection, the spleen was then returned to the abdomen cavity. The abdomen wall was closed using absorbable suture (Dexon) in a continuous suture pattern. The skin will be closed using a 6–0 nylon suture (Dexon) in an interupted suture patter. 0.1 mg/kg buprenorphine will be injected subcutaneously after operation. 1.0 mg/kg atipamezole will be injected ip to reverse dexmedetomidine. Animals were kept in a clean cage. The research staff gave warm fluid subcutaneously (1 ml/100 g body weight) and also put a petri dish with water soaked food on the cage floor. After one week, animals were anesthetized with isoflurane in a desiccator jar in the appropriate fume hood. The sutures on the body wall were removed. Mice were monitored daily for adverse effects. Six weeks after intrasplenic injection, the mice were sacrificed and autopsy performed to check for formation of liver metastases.

### Statistical analysis

All results were expressed as mean ± SD. Two-group comparison was assessed by nonpaired, two tailed Student’s t test. The difference between multiple groups was assessed by ANOVA.

## Results

### 1. FoxM1 and PTTG1 are concordantly expressed in colorectal cancer

**Fig. 1:**
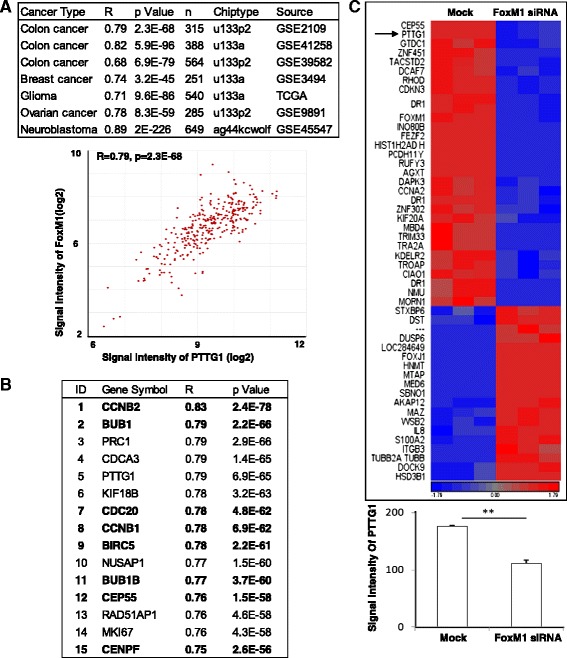
Correlation between FoxM1 and PTTG1 expression in colorectal cancer. **a** FoxM1 closely correlates to PTTG1 expression in 7 tumor microarray datasets (upper table). Expression levels of FoxM1 and PTTG1 were obtained from a colon cancer microarray dataset (GSE2019, *n* = 315) and plotted in lower figure. **b** The top 15 genes associated with FoxM1 expression in GSE2019 dataset. Known FoxM1 target genes were bolded. PTTG1 is ranked as fifth. **c** Differentially expressed genes in BT-20 breast cancer cells transfected with mock or FoxM1 siRNA. The microarray data were standardized (shift genes to means of zero and scale to standard deviation of one). Two-way Hierarchical Clustering was performed. The heat map (upper figure) shows that 49 genes were differentially expressed including CEP55 and PTTG1. The lower figure indicated that the PTTG1 is statistically down-regulated by FoxM1 knock-down

Multiple microarray datasets were analyzed to determine the correlation between FoxM1 and PTTG1 mRNA levels. The results consistently demonstrated that the mRNA levels of PTTG1 and FOXM1 are highly correlated. The levels of correlation (R = 0.68 ~ 0.89, *p* = 2.1E-226 ~ 9.6E-86) for seven datasets are shown in Fig. 1a, which include three colon cancer datasets (GSE2109, *n* = 315; GSE41258, *n* = 388; GSE39582, *n* = 564), one breast cancer dataset (GSE3494, *n* = 251), one glioma dataset (TCGA, *n* = 403), one ovarian cancer dataset (GSE9891, *n* = 285) and one neuroblastoma dataset (GSE45547, *n* = 649). We further analyzed GSE2109 dataset to determine whether known FoxM1 targeted genes exhibited concordant expression pattern with FoxM1. In the top 15 concordantly expressed genes, 8 are known FoxM1 targets, including CCNB2, BUB1, CDC20, CCNB1, BIRC5, BUB1B, CEP55 and CENPF. PTTG1 was ranked as the fifth (R = 0.79, *P* = 6.9E-65) gene concordantly expressed with FoxM1 (Fig. 1b), suggesting that PTTG1 is a potential target of FoxM1.

We also analyzed microarray dataset derived from BT-20 breast cancer cells transfected with mock or FoxM1 siRNA (GSE2222) [36]. Forty nine genes were differentially expressed between mock and FoxM1 siRNA transfected cells (FDR < 0.05). Centrosomal protein 55 kDa (CEP55), a known target gene of FoxM1, was down-regulated 1.5 folds (*p* = 2.95E-05) in the FoxM1 siRNA transfected cells. PTTG1 was down-regulated 1.8 folds (*p* = 7.40E-05, Fig. 1c).

### 2. FoxM1 regulates the transcription and expression of PTTG1

**Fig. 2:**
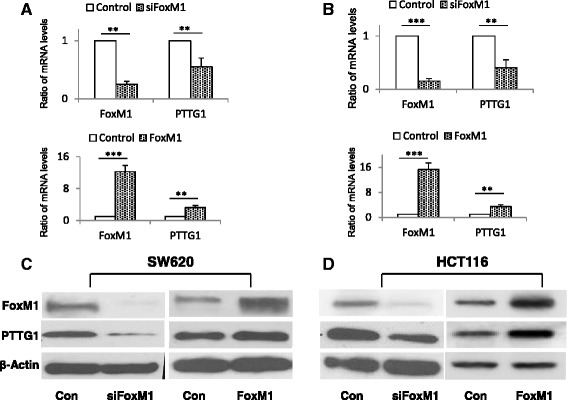
FoxM1 induces PTTG1 mRNA and protein expression in colon cancer cells. FoxM1 siRNA suppressed PTTG1 mRNA expression. SW620 (**a**) and HCT116 (**b**) cells were transfected with negative control siRNA (Con), FoxM1 siRNA (siFoxM1), negative control pcDNA3.1 (Con) or FoxM1 plasmid (FoxM1), respectively. FoxM1 and PTTG1 mRNA levels were detected by real-time PCR; results were normalized with controls and expressed as mean ± SD from triplicate assays. FoxM1 siRNA inhibited, and over-expression increased PTTG1 protein expression. SW620 (**c**) and HCT116 (**d**) cells were transfected with negative control siRNA (Con), FoxM1 siRNA (siFoxM1), negative control pcDNA3.1 (Con) or FoxM1 plasmid (FoxM1), respectively. FoxM1 and PTTG1 protein levels were measured by western blot. All western blot experiments were repeated three times. Results of a representative experiment are shown. *p < 0.05, **p < 0.01, ***p < 0.001

To determine whether FoxM1 modulates PTTG1 expression in colorectal cancer cells, we first examined FoxM1 expression in 61 colorectal cancer cell lines using microarray data derived from CCLE (Cancer Cell Line Encyclopedia) [37]. The results indicate that most colorectal cancer cell lines, including SW620 and HCT116, express abundant FoxM1 mRNA (Additional file 2). SW620 and HCT116 colorectal cancer cells were then transfected with mock or FoxM1 siRNA. As shown in Fig. 2a and b, transfection of FoxM1 siRNA suppressed FoxM1 mRNA by ~87 % in HCT116 and ~70 % in SW620 cells, and reduced PTTG1 mRNA by ~57 % in HCT116 and ~36 % in SW620 cells, respectively. As a result, FoxM1 protein level was reduced by ~99 % in SW620 and ~63 % in HCT116 cells, and PTTG1 protein expression decreased ~75 % in SW620 and ~40 % in HCT116 cells, respectively (Fig. 2c and d). After transfected with FoxM1 expression plasmids, the FoxM1 mRNA levels increased 12.23 fold in SW620 and 15.34 fold in HCT116 cells. The protein levels increased 3.67 folds in SW620 and 4.97 folds in HCT116 cells (Fig. 2c and d). Over-expressed FoxM1 enhanced PTTG1 mRNA expression 3.22 folds and protein expression 1.43 folds in SW620 cells. Similar results (3.4 folds in mRNA levels and 2.23 folds in protein levels) were obtained in HCT116 cells (Fig. 2c and d). These results suggest that FoxM1 indeed enhances PTTG1 transcription.

### 3. FoxM1 binds to PTTG1 promoter

**Fig. 3:**
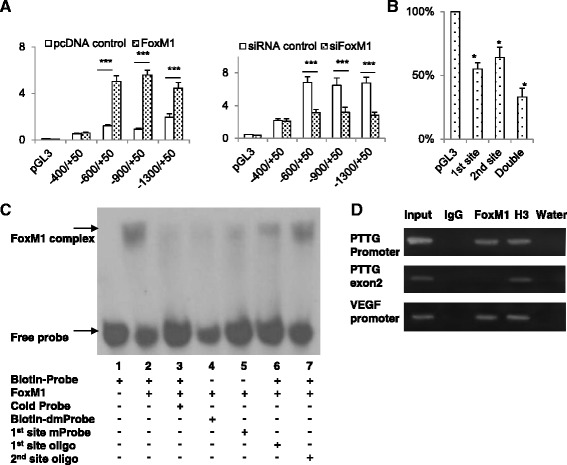
FoxM1 binds to and activates PTTG1 promoter. **a** Luciferase reporter assay. SW620 cells were transfected with pGL3-Basic, pGL3-PTTG1 –1300, −900, −600 or −300/+50 reporter plasmids and co-transfected with pcDNA3.1 control, pcDNA3.1-FoxM1 plasmid (FoxM1), siRNA control or FoxM1 siRNA (siFoxM1), together with Renilla reporter vector. Cells were harvested after 24 h and luciferase activities were examined. After normalizing to Renilla, results are expressed as mean ± SD. Assays were performed in triplicate and experiments were repeated three times. Results of a representative experiment are shown. *p < 0.05, **p < 0.01, ***p < 0.001. **b** FoxM1 binding sites on the -600 bp PTTG1 promoter (pGL3-PTTG1-600) are mutated and these mutants are co-transfected with FoxM1 to evaluate effects of FoxM1 on the activity of mutated promoter (1^st^ site: −391 to −385, 2^nd^ site: −359 to −353 bp of TSS). **c** Biotin-streptavidin EMSA. Representative EMSA shows the bindings of FoxM1 protein with Biotin-Probe in lane 2 (biotin-labeled 60 bp probe, containing both −391 to −385 and −359 to −353 bp wild type FoxM1-binding PTTG1 promoter motifs), with Biotin-dmProbe in lane 4 (biotin-labeled 60 bp probe, containing both −391 to −385 and −359 to −353 bp mutant FoxM1-binding sites) and with 1^st^ site mProbe (biotin-labeled 60 bp probe with −391 to −385 bp mutant FoxM1-binding site, lane 5). Competition assays were performed using a 100-folds excess of cold probe (unlabeled 60 bp wild type probe, lane 3), 1^st^ site oligo (unlabeled 22 bp oligonucleotide only containing −391 to −385 bp wild type FoxM1-binding motif, lane 6) and 2^nd^ site oligo (unlabeled 25 bp oligonucleotide only containing −359 to −353 bp wild type FoxM1-binding motif, lane 7). **d** ChIP assay showing specific endogenous FoxM1 binding to the human PTTG1 promoter. About 500 bp length chromatin DNA in HCT116 cells were immunoprecipitated with FoxM1 antibody, negative control IgG, positive control histone H3 antibody (H3) and water blank control as indicated. Enrichment of chromatins was obtained with primers targeting PTTG1 promoter but not with primers targeting PTTG1 exon 2. The experiment was repeated twice. As VEGF is a known target of FoxM1, primers targeting VEGF promoter were included as a positive control

GeneQuest (Lasergene7.1) identified two potential FoxM1-binding motifs, i.e. 5’-TATTTAT-3’and 5’-TAAATAA-3’, located at −391 to −385 (1^st^ site) and −359 to −353 bp (2^nd^ site) upstream of the translation initiation site of PTTG1. Four PTTG1 promoter fragments covering 5’ upstream −1300, −900, −600 and −300 to +50 bp were cloned into pGL3-basic luciferase plasmid. These luciferase plasmids were co-transfected with FoxM1 or control plasmid into colorectal cancer cells, and promoter activity measured. As shown in Fig. 3a, all three constructs induced transcriptional activities in response to FoxM1 co-transfection in both cell lines. Similarly, the luciferase plasmids were co-transfected with FoxM1 siRNA and corresponding control siRNA. The −1300, −900 and −600 bp PTTG1 promoter activity was significantly inhibited after co-transfected with FoxM1 siRNA (Fig. 3a), suggesting that the FoxM1 responsive elements locate between −600 to −300 bp region. We mutated the FoxM1 binding sites of the –600 bp PTTG1 promoter in the pGL3-basic plasmid and evaluated FoxM1 effects on the mutated promoters. Mutation of 1^st^ site leads to reduction of 45 % of promoter activity. Mutation of 2^nd^ site leads to reduction of 34 %. Loss of both FoxM1 binding sites leads to a reduction of 77 % promoter activity when co-transfected with FoxM1 (Fig. 3b).

Next, we carried out a biotin-streptavidin EMSA to verify the interaction between FoxM1 protein and heptamer motifs at −391 to −385 and −359 to −353 bp of the PTTG1 promoter. A 60 bp biotin-labeled probe containing two FoxM1-binding motifs on the human PTTG1 promoter was synthesized and the binding with FoxM1 was examined. As shown in Fig. 3c, the incubation of labeled probe and FoxM1 protein resulted in the appearance of a DNA-protein complex (lane 2). The DNA-protein binding was abolished by adding cold competitive probe (lane 3), strongly inhibited by adding the 60 bp mutant probe with the first site (−391 to −385 bp) mutated (lane 5) and the 20 bp mutant oligonucleotide with the first site mutated (lane 6), and slightly reduced by adding the 20 bp mutant oligonucleotide with the second site (−359 to −353 bp) mutated (lane 7). We also used double sites mutated probe as negative control, which exhibited diminished DNA-protein interaction band (lane 4). These results indicated that both FoxM1-binding sites in the PTTG promoter participates in PTTG1 transcriptional regulation by FoxM1, and directly bind FoxM1 protein. However, we failed to generate supershifts when adding FoxM1 antibody to the EMSA reaction, which might be due to lack of a qualified FoxM1 antibody for EMSA.

As FoxM1 protein is in a native state in EMSA assay but in a denatured state in ChIP, FoxM1 antibody not suitable for EMSA may be able to recognize the denatured FoxM1 in the ChIP experiments. We then performed ChIP assay to determine whether FoxM1 binding to PTTG1 promoter. About 10^7^ HCT116 cells were fixed and chromatin DNA sonicated into about 200–500 bp fragments. Equal amounts of chromatin DNA were incubated separately with IgG, FoxM1 antibody, histone H3 antibody or water. The captured chromatin DNA fragments were analyzed using PCR by three primer pairs targeting different region (PTTG1 -391 to −353, PTTG1 exon2, VEGF promoter). As shown in Fig. 3c, DNA enriched by FoxM1 antibody was positive for primers targeting PTTG1 promoter −391 to −353 bp region, indicating that FoxM1 specifically binds to the PTTG1 promoter. Primers targeting PTTG1 exon2 were used as a negative control, which produced a positive amplification in the input sample and a negative amplification in the FoxM1 ChIP sample. Primers targeting VEGF promoter were used as a positive control and produced a positive signal in both input and FoxM1 ChIP sample. As the two FoxM1 binding sites are only 30 bp away from each other, we are not able to discriminate the binding difference of FoxM1.

### 4. Attenuate PTTG1 and FoxM1 expression suppress migration, invasion and metastasis of colon cancer cells

**Fig. 4:**
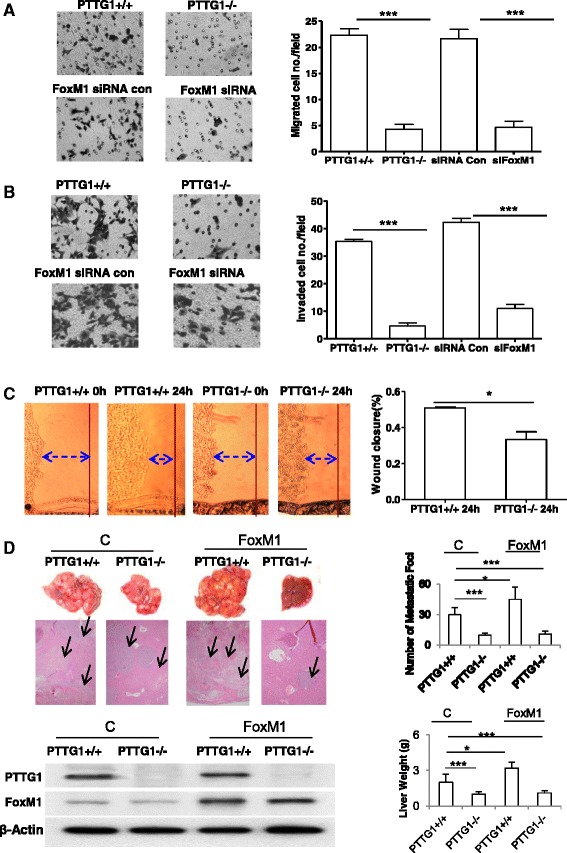
PTTG1 knock-out and FoxM1 knock-down suppress colon cancer cell migration, invasion and liver metastasis. **a**, **b** *In vitro* migration (**a**) and invasion (**b**) assays in HCT116 cells. Left panel: representative migration and invasion photographs of PTTG1^+/+^, PTTG1^−/−^, control siRNA- and FoxM1 siRNA (siFoxM1)-transfected cells. Right panel: quantification of migrated and invaded cells per field at × 40 magnification. Experiments were repeated three times. **c** Wound-healing assay in FoxM1 over expressing PTTG1^+/+^ and PTTG1^−/−^ HCT116 cells. The cells were allowed to form monolayers in a 6-well plate. A wound was created by a pipette tip. The healing of the wounds was monitored under microscope. Pictures were taken at different time points. Left panel: representative migration images of FoxM1 over expressing PTTG1^+/+^ and PTTG1^−/−^ HCT116 cells. Right panel: wound closure percentage was determined by subtracting values obtained at 0 h from 24 h. Experiments were repeated three times. **d** FoxM1 over expressing PTTG1^+/+^ or PTTG1^−/−^ HCT116 cells were injected into spleen to induce liver metastasis. Left panel: representative images of metastatic liver tumor derived from mice injected with control cells and FoxM1 overexpressing cells. Histochemistry and western blots indicated that the metastatic foci are derived from the injected cells and FoxM1 was overexpressed in the FoxM1 overexpressing group. Right panel: summary of metastatic liver foci numbers and liver weight. All data above are presented as the mean ± SD. *p < 0.05, **p < 0.01, ***p < 0.001

High PTTG1 is associated with invasiveness [38]. *In vitro* cell migration and invasion assays indicated that PTTG1 knock-out HCT116 cells exhibited impaired migration and invasion (Fig. 4a and b). The migration of PTTG1^−/−^ HCT116 cells decreased about 80.6 % (*P* < 0.001) and the invasion ability decreased about 86.8 % (*P* < 0.001) compared with those of PTTG1^+/+^ control cells (Fig. 4a and b). In wound-healing assay, PTTG1 knock-out caused 34.3 % (*P* < 0.05) decrease in cell migration compared with those of PTTG1 wild-type HCT116 cells (Fig. 4c). Transfection of FoxM1 siRNA in HCT116 cells also resulted in a significant decrease of migration (78.4 %; *P* < 0.001) and invasion (74 %; *P* < 0.001), compared with cells transfected with siRNA control (Fig. 4a and b). We also investigated whether forced expression of FoxM1 would increase cell migration and invasion. Surprisingly, transfection of FoxM1 plasmids did not further enhance the migration and invasion of WT or PTTG1^−/−^ HCT116 cells (Data not shown), which could due to a high baseline expression of FoxM1 in the tested cells.

To investigate the role of PTTG1 and FoxM1 in colon cancer metastasis, we stably expressed FoxM1 in PTTG1^+/+^ and PTTG1^−/−^ HCT116 cells, and implanted these cells into the spleen of nude mice to induce liver metastases. Mice were killed 6 weeks after intrasplenic injection. In the PTTG1^+/+^ control group without FoxM1 over expression, 8 out of 10 developed liver tumors (15–34 metastatic loci). The PTTG1^−/−^ control group without FoxM1 over expression, 5 out of 10 developed live metastases (4–14 metastatic loci). All 10 mice (100 %) in the PTTG1^+/+^ FoxM1 overexpressing group developed liver tumors (35–58 metastatic loci), while 4 out of 10 mice in the PTTG1^−/−^ FoxM1 group exhibited small liver tumors (5–13 metastatic loci). The overall liver weights in the PTTG1^−/−^ implanted mice were much less than these derived from PTTG1^+/+^ group. Overexpression of FoxM1 enhanced liver metastases of the PTTG1^+/+^ HCT116 cells but not these of the PTTG1^−/−^ cells. Histochemistry indicated that the metastatic foci (marked by arrow head) are derived from colorectal cancer (Fig. 4d). Western blots confirmed the enhanced FoxM1 expression in the metastatic foci derived from FoxM1 stably expressed cells. The results support the above *in vitro* cell migration and invasion findings and indicate that PTTG1 knock out attenuates FoxM1-induced colon cancer metastasis.

### 5. FoxM1-PTTG1 regulates Wnt antagonist DKK1

**Fig. 5:**
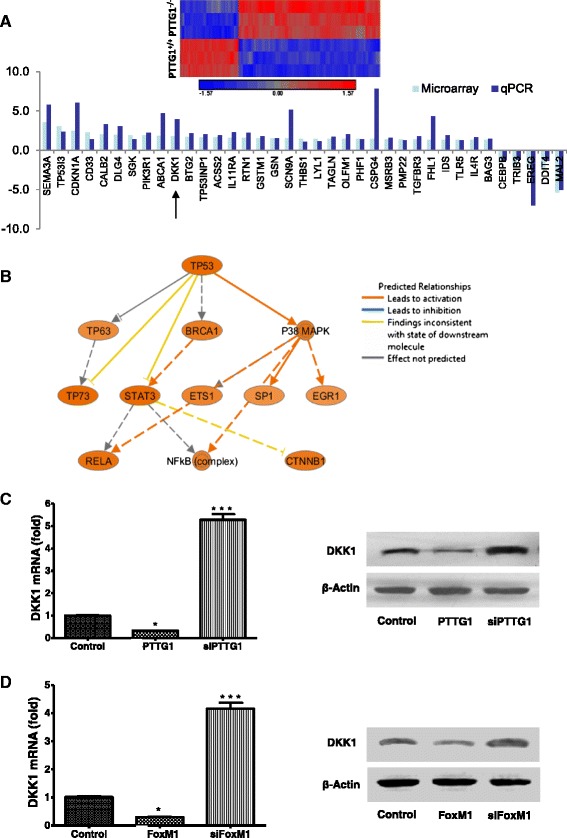
Genes regulated by PTTG1. **a** Differentially expressed genes in WT and PTTG1^−/−^ HCT116 cells. Upper: heat map of 662 differentially expressed genes (FDR < 0.05) between WT and PTTG1^−/−^ HCT116 cells. Lower: 37 out of 40 genes were consistent between microarray and qPCR. Results of qPCR were calculated using the ∆∆Ct method with GAPDH as an internal control. **b** Signaling network analysis of 662 differentially expressed genes revealed a network involve p53 and CTNNB1. **c** PTTG1 regulates DKK1 expression. HCT116 cells were transfected with pcDNA3.1 and negative control siRNA (control), pcDNA3.1 and PTTG1 siRNA (siPTTG1), control siRNA and pcDNA3.1-PTTG1 (PTTG1), respectively. Left: FoxM1, PTTG1 and DKK1 mRNA levels were detected by real-time qPCR. Right: FoxM1, PTTG1 and DKK1 protein levels were detected by western blot. **d** FoxM1 regulates PTTG1 and DKK1 expression. HCT116 cells were transfected with pcDNA3.1 and negative control siRNA (control), pcDNA3.1 and FoxM1 siRNA (siFoxM1), control siRNA and pcDNA3.1-FoxM1 (FoxM1), respectively. Left: FoxM1, PTTG1 and DKK1 mRNA levels were detected by qPCR. Right: FoxM1, PTTG1 and DKK1 protein levels were detected by western blot. All qPCR were performed in triplicates and normalized to GAPDH. β-actin was used as an internal control for western blot. Results from three experiments were expressed as mean ± SD. Western blot experiments were repeated three times. Results of a representative experiment are shown. *p < 0.05, **p < 0.01, ***p < 0.001

Using Illumina HumanHT-12_V4 microarrays, we compared the gene expression profiles of PTTG1^+/+^ and PTTG1^−/−^ HCT116 cells (GSE58863). We identified 662 significantly differentially expressed genes (FDR < 0.05, Fig. 5a). We randomly selected 40 genes for validation using qPCR, and found that 37 genes were consistent up or down regulated between qPCR and microarray analysis (Fig. 5a), including 32 up-regulated genes (including SEMA3A, TP53I3, CDKN1A, CD33, CALB2, DLG4, SGK, PIK3R1, ABCA1, DKK1, BTG2, TP53INP1, ACSS2, IL11RA, RTN1, GSTM1, GSN, SCN9A,THBS1, LYL1, TAGLN, OLFM1, PHF1, CSPG4, MSRB3, PMP22, TGFBR3, FHL1, IDS, TLR5, IL4R, and BAG3) and 5 down-regulated genes (including CEBPB, TRIB3, EREG, DDIT4 and MAL2).

To obtain insight into the role of PTTG1 in colon cancer cells, we performed ingenuity pathway analysis (IPA) using the differentially expressed genes present in PTTG1^−/−^ HCT116 cell. The network analysis highlighted an enhancement of TP53 signaling pathway. It was predicted that the enhanced Tp53 further modulates STAT3 to attenuate CTNNB1, a key component of Wnt pathway (Fig. 5b). As DKK1, an inhibitor of the Wnt signaling pathway, is induced by p53 [39], we seek to determine whether DKK1 mediates the FoxM1 and PTTG1 effects. DKK1 expression was examined in HCT116 cells when FoxM1 or PTTG1 are over-expressed or knock-down. The DKK1 mRNA and protein levels decreased ~67 % and ~50 % (Fig. 5c, column 2), respectively, in response to PTTG1 over-expression and increased over 5.28 folds and 2.5 folds (Fig. 5c, column 3), respectively, as a result of PTTG1 knock-down. Meanwhile, the DKK1 mRNA and protein levels reduced ~71 % and ~45 % under FoxM1 over-expression (Fig. 5d, column 2) and increased 4.1 -folds and 1.8 folds in response to FoxM1 knock-down (Fig. 5d, column 3).

## Discussion

Bioinformatic analyses indicated that FoxM1 and PTTG1 are concordantly expressed in human colorectal cancer tissues, suggesting PTTG1 is a potential target of FoxM1. We further presented evidence that FoxM1 activates PTTG1 transcription through binding to PTTG1 promoter. Our results indicated that forced over expression of FoxM1 up-regulates PTTG1. FoxM1 binds to DNA with a preference for a consensus 5’-A-T/C-AAA-T/C-AA-3’ recognition sequence [40] and there are two potential FoxM1 binding sites on the PTTG1 promoter. Luciferase reporter assay confirmed the transcriptional regulatory activity of FoxM1 on PTTG1 promoter. EMSA indicated that FoxM1 binds to both FoxM1 binding sites on the PTTG1 promoter. The binding of FoxM1 on the PTTG1 promoter is confirmed by ChIP assay.

The functions of FoxM1 and PTTG1 in regulating cell migration and invasion were surveyed. Boyden chamber assay indicated that FoxM1 and PTTG1 siRNAs attenuated migration and invasion of HCT116 and SW620 colorectal cancer cells. Wound-healing assay further suggested that PTTG1 mediates FoxM1 effects in regulating cell migration. The *in vivo* tumor xenografts also provided evidence that PTTG1 participates in the FoxM1-mediated liver metastases of colon cancer.

FoxM1 plays a central role in regulating many cellular functions, including cell proliferation, cell survival, and immortalization, and is critical to carcinogenesis and metastasis of cancers, including gastric cancer, colorectal cancer, pancreatic cancer, breast cancer, non-Hodgkin’s lymphoma, glioma and malignant peripheral nerve sheath tumors [26, 27, 41, 42]. Over-expression of FoxM1 involved in cell-cycle progression, invasion and angiogenesis, epithelial-to-mesenchymal transition (EMT) directly through up-regulating of its target genes, e.g. CDC25B, CCNB1, AURKB, PLK1, CENPA, CENPB, MYC, SKP2, MMP2 and VEGF. In present study, we identified PTTG1 as a FoxM1 target gene in colon cancer, which provides a mechanism of FoxM1 involved in the metastasis of malignances [26, 43–45]. We discovered that FoxM1 directly bound to the promoter region of PTTG1 gene, which is inconsistent with the previous report [46]. We also provide evidence showing that FoxM1 activates PTTG1 transcription. PTTG1 is required for FoxM1 to promote colorectal cancer cell migration and invasion. Identification of PTTG1 as a FoxM1 target gene provides further evidence linking FoxM1 function to carcinogenesis and progression.

Up-regulation of PTTG1 has been correlated with aggressive disease and poor prognosis in hepatocellular carcinoma, prostate cancer, esophageal cancer, glioma, thyroid cancer and colorectal cancer [13–19]. Previous studies showed that PTTG1 was directly regulated by estrogen, insulin, basic fibroblast growth factor, epidermal growth factor, β-catenin/transcription factor, Rb/E2F1 pathways, STAT3, etc., and consequently involved in multiple steps of tumor progression including tumorigenesis, invasiveness, metastasis, and angiogenesis [11, 20–24, 47]. Our study demonstrated a novel FoxM1-PTTG1 relation and implied a new mechanism of PTTG1 involving tumor progression.

DKK1, as a Wnt antagonist, shows wide and complex effects on cell proliferation and differentiation. The distinct effects of DKK1 seem to depend on the cell type, which agrees with the different effects of Wnt/β-catenin signaling [48]. In colon cancer, DKK1 was found down-regulated, indicating the loss of a negative feedback control of the Wnt/β-catenin pathway in this neoplasia [49]. Our microarray data showed that DKK1 was up-regulated in PTTG1^−/−^ cells and the further *in vitro* assays indicated that DKK1 was down-regulated by over expressing either FoxM1 or PTTG1. Our results suggest that abnormal up-regulated FoxM1-PTTG1 pathway may promote colon cancer through suppressing DKK1 and thereby enhancing the Wnt pathway. In addition, the IPA analysis of PTTG1-regulated genes indicated an enhanced TP53 pathway, which are consistent with previous report [50]. DKK1 has been found induced by TP53 [39], which suggested that FoxM1-PTTG1 pathway may regulate DKK1 through modulating TP53 pathway. PTTG1 has been shown to be a direct target gene of β-catenin [51]. PTTG1 may therefore provide a positive feedback to enhance its own transcription by enhancing WNT pathway through suppressing DKK1.

In summary, we provide evidence that (1) FoxM1 regulates PTTG1 transcription through binding to PTTG1 promoter; (2) FoxM1 or PTTG1 knockdown attenuate colorectal cancer cell migration, invasion and metastasis; (3) FoxM1 activates PTTG1, which subsequently suppresses Wnt antagonist, DKK1. These findings expand our knowledge on regulatory mechanisms underlying tumor PTTG1 abundance. In the ongoing search for molecular points of therapeutic intervention for various malignancies, both PTTG1 and FoxM1 have been considered as promising and attractive targets for cancer therapy. The FoxM1-PTTG1 pathway we have identified will help to define the proper therapeutic window in which targeting FoxM1 or PTTG1 can achieve clinical benefits. However, more studies are needed to further understand the precise mechanisms of the FoxM1 and PTTG1 involved in different cellular contexts and tumor types, and in response to different types of damage.

## Conclusions

The results indicate that PTTG1 is a FoxM1 targeted gene. FoxM1 binds to PTTG1 promoter to promote PTTG1 transcription, and FoxM1-PTTG1 pathway promotes colorectal cancer migration and invasion.

## Additional files

Additional file 1:
**Real time qPCR primers.** (XLS 16 kb) Additional file 2:
**FoxM1 mRNA levels in 61 colorectal cancer cell lines.** (XLS 11 kb)
